# Characterization of the unique oral microbiome of children with Down syndrome

**DOI:** 10.1038/s41598-022-18409-z

**Published:** 2022-08-19

**Authors:** Chieko Mitsuhata, Nao Kado, Masakazu Hamada, Ryota Nomura, Katsuyuki Kozai

**Affiliations:** 1 Department of Pediatric Dentistry, Graduate School of Biomedical and Health Sciences, Hiroshima University, 1-2-3 Kasumi, Minami-ku, Hiroshima, 734-8553 Japan; 2 Department of Oral and Maxillofacial Surgery II, Osaka University Graduate School of Dentistry, 1-8 Yamada-oka, Suita, Osaka, 565-0871, Japan

**Keywords:** Microbiology, Bacteriology

## Abstract

Down syndrome creates an abnormal oral environment, including susceptibility to periodontal disease at a young age, but there are no detailed studies of the oral microbiome in children with Down syndrome. In this study, we performed a comprehensive analysis of the oral bacteria of 40 children with Down syndrome and 40 non-Down syndrome children. Microbial DNA was extracted from dental plaque specimens and the V4 hypervariable region of the bacterial 16S rRNA gene was analyzed using the MiSeq platform. There were significant differences between the Down syndrome and non-Down syndrome groups in mean numbers of operational taxonomic units, and α- and β-diversity (*P* < 0.05). Interestingly, significant differences in α- and β-diversity between the two groups were only observed in subjects with gingival inflammation, but not in those without gingival inflammation (*P* < 0.05). Taxonomic analysis at the genus or species levels showed significant differences in relative abundance levels of certain bacteria between the Down syndrome and non-Down syndrome groups, including *Corynebacterium*, *Abiotrophia* and *Lautropia* (*P* < 0.05). These results suggest that children with Down syndrome may have a unique oral microbiome that could impact the development of dental diseases common in people with the syndrome.

## Introduction

Down syndrome is a chromosomal disorder involving an extra full or partial copy of chromosome 21 and occurring at a rate of approximately 1 in 600–1000 births^1^. Its characteristics include distinctive facial features, heart malformations, intellectual disability, and deficiencies in the immune and endocrine systems^1^. The reported relationship between Down syndrome pathology and several genes encoded on chromosome 21 is attributed to multiple factors, which include changes in expression levels of genes throughout the genome exerted through multiple intergenic networks on chromosome 21, as well as changes in the structure of chromatin^2^.

A variety of characteristic findings on the oral cavity of individuals with Down syndrome have been reported, including oral breathing, decreased eating function due to relaxation of the narrow palate/perioral muscles, lower muscle tone in the tongue, macrognathia, abnormal tooth morphology, occlusal abnormalities, and increased risk of periodontal disease^1^. Among these, periodontal disease often occurs at an early age and is more severe in people with Down syndrome^3–5^. Furthermore, colonization of oral bacteria established in childhood may exacerbate periodontal disease^6^. Recently, a study focusing on the oral microbiome of adults with Down syndrome was reported^7^, but the oral microbiome of children with Down syndrome remains unknown.

More than 800 bacterial species inhabit the oral cavity, of which 70% are unculturable^8^. These organisms coexist in the oral cavity in a mutualistic manner, and changes in their balance can affect health and even cause disease. Comparisons of oral bacteria in children with Down syndrome with periodontal disease and dental caries have been performed using PCR, DNA–DNA hybridization, and measurements of specific antibody titers^6,9,10^. However, these methodologies focus on specific, culturable bacteria. Relationships between changes in the oral flora with disease and the systemic and local characteristics of Down syndrome necessitate a comprehensive analysis of the oral flora in this population.

In the present study, we investigated and compared the microbiomes in the oral cavity of children with and without Down syndrome using next generation sequencing to identify bacteriologic factors that may affect oral conditions that are common in people with Down syndrome.

## Results

### Clinical characterization of oral health in children with and without Down syndrome

We enrolled 40 children with Down syndrome (18 boys and 22 girls; mean age, 10.20 ± 4.45 years; age range, 2–18 years) and 40 non-Down syndrome children as controls (18 boys and 22 girls; mean age, 9.31 ± 4.34 years; range, 2–18 years) into the study (Table 1). The clinical characteristics of oral health in the two groups are shown in Table 2. Numbers of permanent teeth, the decayed, missing and filled teeth (DMFT) index, and the prevalence of dental caries were significantly lower in the Down syndrome group compared with the control group (*P* < 0.05). The mean plaque index was 2.13 in the Down syndrome group and 1.93 in the control group, with no significant difference between the two groups. The gingival index (GI) in the permanent teeth was significantly higher in the Down syndrome group than the control group (*P* < 0.01). Bleeding on probing (BOP) was also significantly higher in participants with Down syndrome compared with controls, in both primary and permanent dentitions.

**Table 1:** Number of study subjects.

Clinical characteristics	Down syndrome (n = 40)	Control (n = 40)
**Total number**	40 (10.20 ± 4.45)	40 (9.31 ± 4.34)
Gender		
Male	18 (10.13 ± 4.45)	18 (8.92 ± 4.15)
Female	22 (10.20 ± 4.45)	22 (9.71 ± 4.56)
Dentition stage		
Primary	12 (4.92 ± 1.80)	9 (3.85 ± 1.09)
Mixed	17 (10.31 ± 2.57)	19 (9.16 ± 1.81)
Permanent	11 (15.30 ± 1.86)	12 (14.56 ± 2.40)
Gingival inflammation		
Inflamed	33 (10.93 ± 4.25)	19 (9.83 ± 3.28)
Non-inflamed	7 (6.36 ± 3.74)	21 (9.46 ± 4.90)

Numbers in parentheses indicate mean ± SD of age.

**Table 2:** Characteristics of study subjects.

Clinical characteristics	Down syndrome (n = 40)	Control (n = 40)	*P*-value
**Dental examination**			
DMFT/dmft index			
Total	0.65 ± 1.74	0.83 ± 1.14	0.054
Primary	0.08 ± 0.28	0.00 ± 0.00	0.75
Mixed	0.94 ± 2.26	0.84 ± 1.23	0.56
Permanent	0.82 ± 1.59	1.42 ± 1.04	**0.047**
**Dental caries prevalence (%)**			
Total	20.0	42.5	**0.003**
Primary	8.3	0	0.37
Mixed	23.5	36.8	0.34
Permanent	27.3	83.3	**0.007**
**PLI**			
Total	2.13 ± 0.84	1.93 ± 0.69	0.19
Primary	1.75 ± 0.92	1.56 ± 0.50	0.62
Mixed	2.18 ± 0.71	2.11 ± 0.72	0.79
Permanent	2.45 ± 0.78	1.92 ± 0.64	0.08
**PD (mm)**			
Total	2.85 ± 0.73	2.63 ± 0.53	0.17
Primary	2.58 ± 0.50	2.33 ± 0.47	0.34
Mixed	2.94 ± 0.54	2.79 ± 0.52	0.51
Permanent	3.00 ± 1.04	2.58 ± 0.47	0.35
**GI**			
Total	1.78 ± 0.52	1.38 ± 0.66	**0.002**
Primary	1.58 ± 0.64	1.11 ± 0.57	0.11
Mixed	1.88 ± 0.32	1.63 ± 0.58	0.3
Permanent	1.82 ± 0.57	1.17 ± 0.64	**0.01**
**BOP (%)**			
Total	82.5	47.5	**0.001**
Primary	66.7	22.2	**0.024**
Mixed	88.2	68.4	0.31
Permanent	90.9	33.3	**0.007**

*DMFT/dmft index* Decayed, Missing, and Filled Teeth/decayed, missing, and filled teeth index, *PLI* Plaque Index, *PD* Probing Depth, *GI* Gingival Index, *BOP* Bleeding on Probing.

Values in DMFT/dmft index, PLI, PD, GI indicate mean ± SD.

The Chi-squared test (Fisher’s exact probability test for dentition comparisons) was used to compare the prevalence of caries and BOP. The Mann–Whitney *U* test was used to analyze the DMFT/dmft index, PLI, PD, and GI. Bold type in *P*-value indicates a significant difference (*P* < 0.05).

### Number of reads and operational taxonomic units (OTUs) in dental plaque

**Figure 1:**
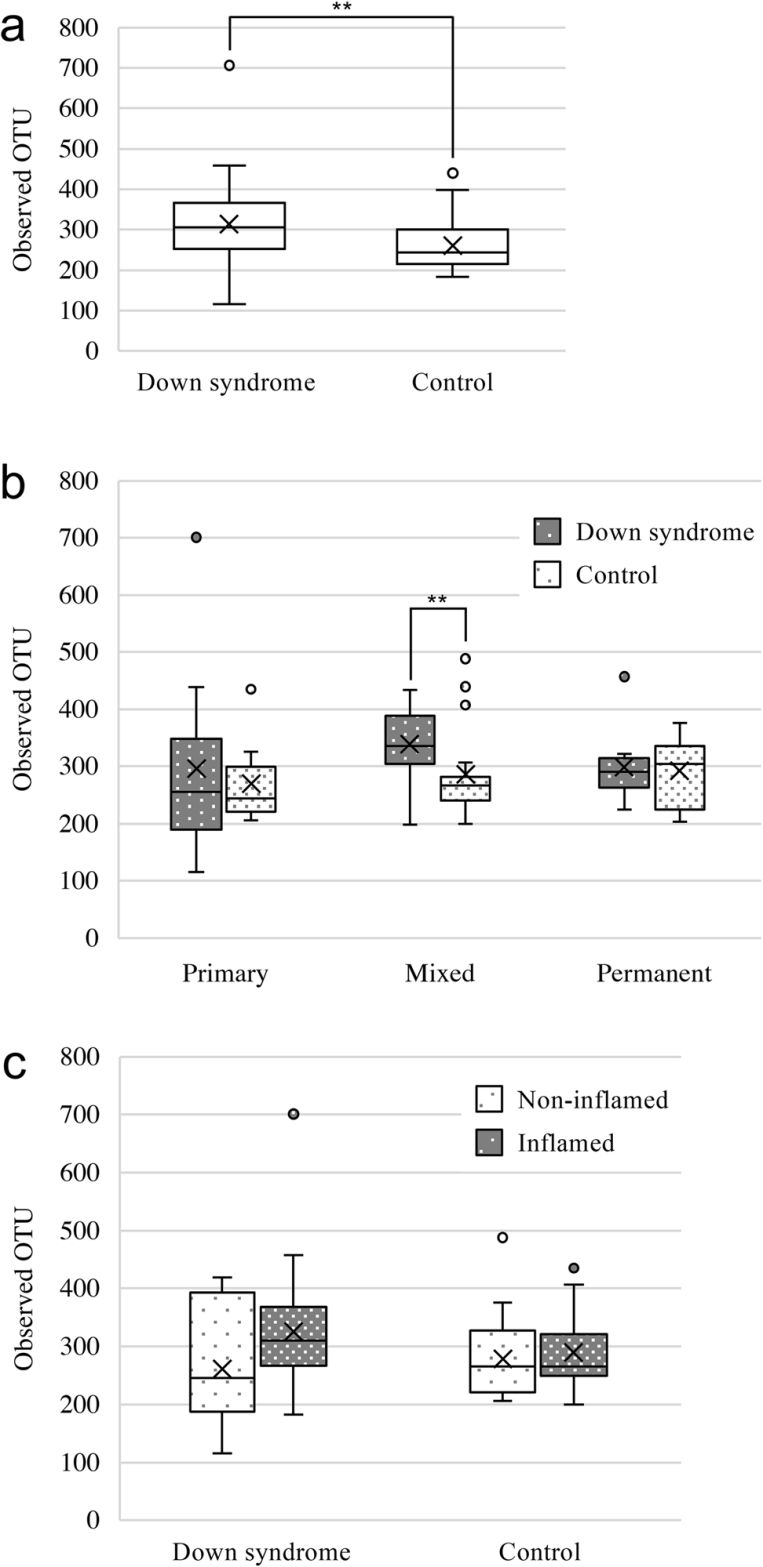
Comparisons of the numbers of operational taxonomic units (OTUs) in dental plaque samples from the Down syndrome and the control groups. Comparisons between the two groups were made for (**a**) all participants, (**b**) dentition stage, and (**c**) gingival inflammation status. Whiskers indicate maximum and minimum values, boxes indicate the interquartile range, X indicates the mean value, and circles indicate outliers. **P < 0.01 by the Mann–Whitney *U* test and Kruskal–Wallis test (pairwise).

Next generation sequencing of the V4 hypervariable region of the bacterial 16S rRNA gene in samples of dental plaque from the two groups yielded a total of 8,424,278 reads, with a mean number of reads/sample of 105,303. The total number of OTUs obtained from all samples was 10,113 (including overlaps between samples). The mean OTU number was significantly higher in the Down syndrome group (314) than the control group (260; *P* < 0.01) (Fig. 1a). When three dentition periods of the two groups were compared, the number of OTUs among participants with mixed dentition was significantly higher in the Down syndrome group than the control group (*P* < 0.01) (Fig. 1b). Gingival inflammation status did not impact the numbers of OTUs within each group (Fig. 1c).

### α-Diversity

**Figure 2:**
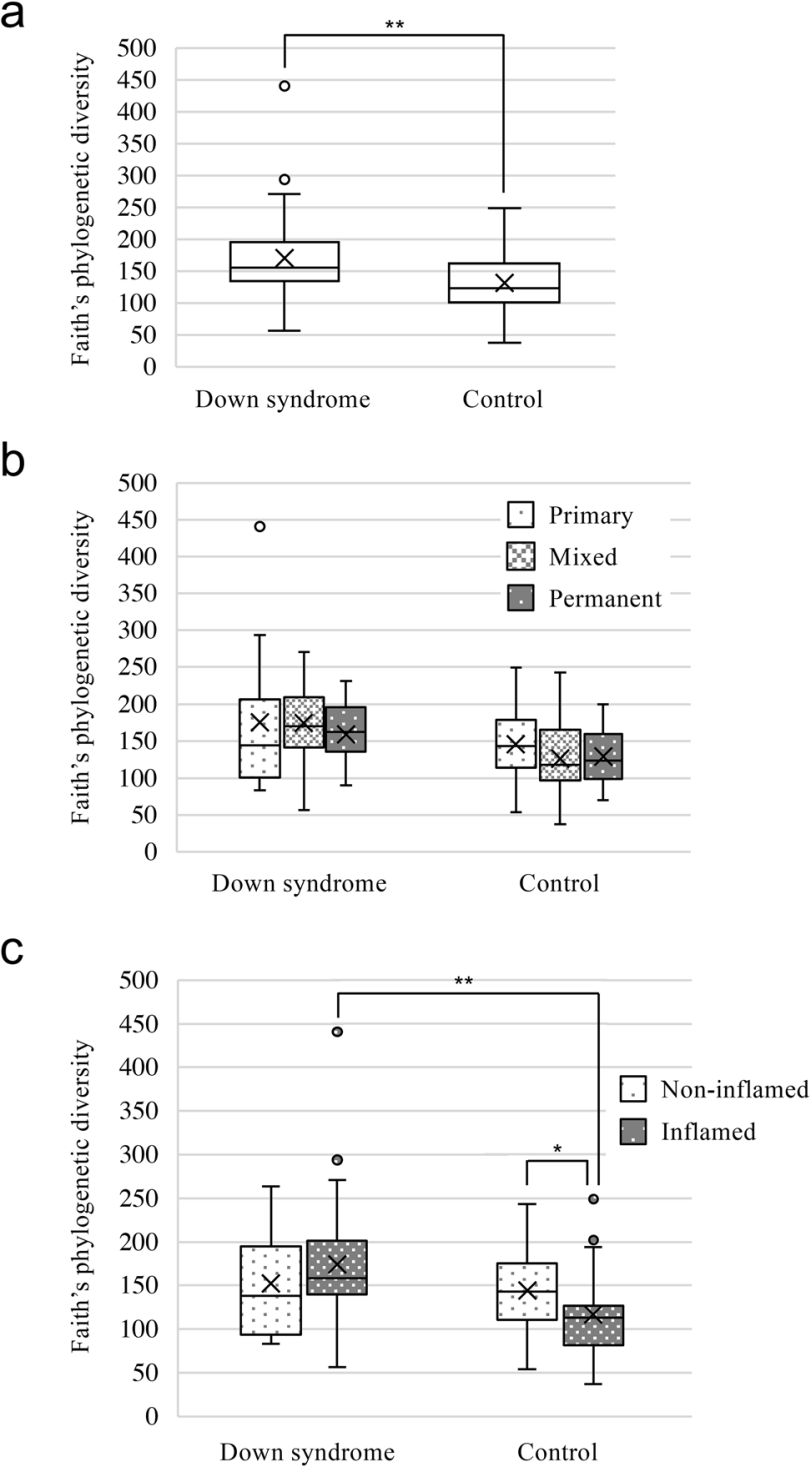
Faith’s phylogenetic diversity analysis of α-diversity in the Down syndrome and the control groups. Comparisons between the two groups were made for (**a**) all participants, (**b**) dentition stage, and (**c**) gingival inflammation status. Whiskers indicate maximum and minimum values, boxes indicate the interquartile range, X indicates the mean value, and circles indicate outliers. *P < 0.05 and **P < 0.01 by the Kruskal–Wallis test (pairwise).

Faith’s phylogenetic diversity analysis revealed that α-diversity was significantly higher in the Down syndrome group compared with the control group (*P* < 0.01) (Fig. 2a). There were no significant differences in α-diversity between the Down syndrome and the control groups among participants at three different stages of dentition (Fig. 2b). In the control group, α-diversity values were significantly lower in participants with gingival inflammation compared to those without inflammation (*P* < 0.01) (Fig. 2c). Among participants with gingival inflammation in the two groups, α-diversity was significantly higher in the Down syndrome group than the control group (*P* < 0.01).

### β-Diversity

**Figure 3:**
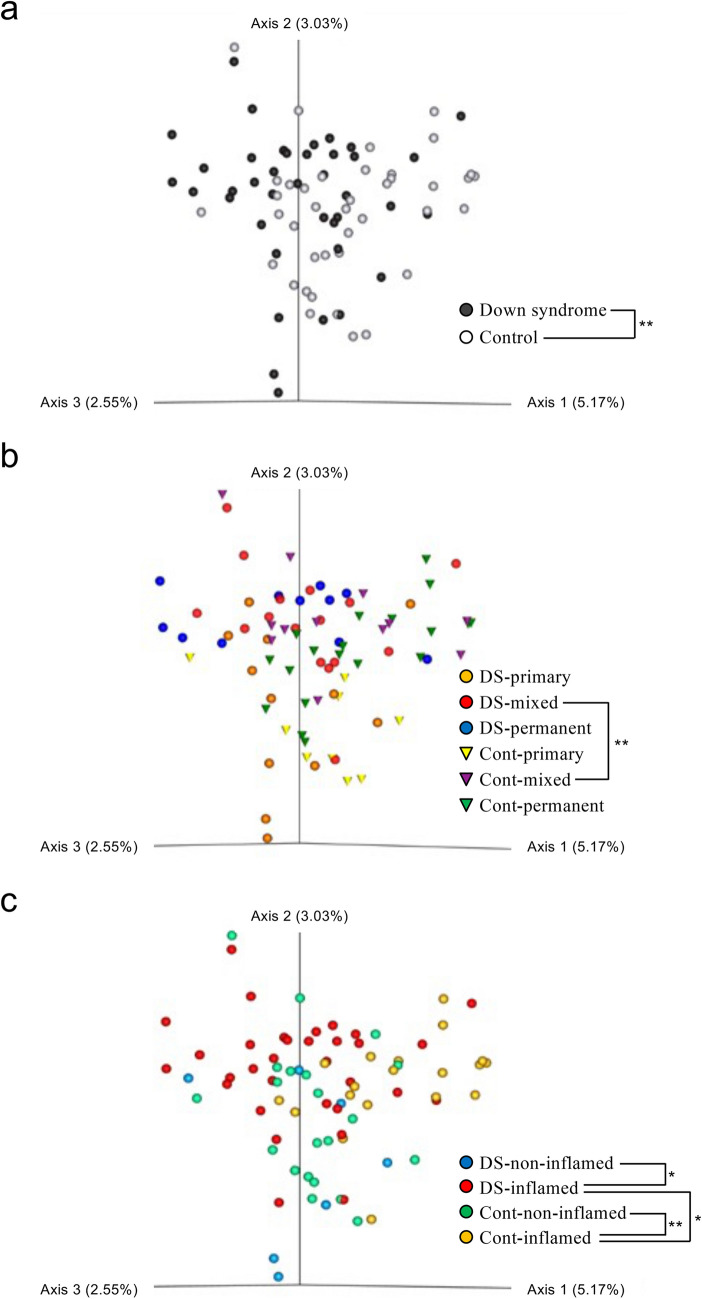
PERMANOVA analysis of β-diversity in the Down syndrome (DS) and control (Cont) groups. Comparisons between the two groups were made for (**a**) all participants, (**b**) dentition stage (mixed, permanent and primary), and (**c**) gingival inflammation status. The contribution rate of each Axis (1–3) in the principal coordinate analysis is shown in parentheses. In this analysis, Axis 1 (3.03%) + Axis 2 (5.17%) + Axis 3 (2.55%) = 10.75%, which is considered to reflect approximately 10% of all of the information. *P < 0.05 and **P < 0.01 by PERMANOVA analysis of the unweighted UniFrac distance.

We analyzed β-diversity using a principal coordinate analysis of the unweighted UniFrac distance, and compared similarities in the oral microflora in the two groups. When inter- and intra-sample distances were compared between the Down syndrome and the control groups, a significant difference was observed in the inter-sample distance (*P* < 0.01) (Fig. 3a). In comparisons of the dentition stage subgroups, we only found a significant difference in β-diversity between the Down syndrome and the control participants with mixed dentition (*P* < 0.01) (Fig. 3b). There were also significant inter-group differences in β-diversity with and without gingival inflammation within each group (*P* < 0.05), and a significant difference between the Down syndrome and the control groups among participants with inflammation (*P* < 0.05) (Fig. 3c).

### Taxonomic analysis at the phylum level

**Figure 4:**
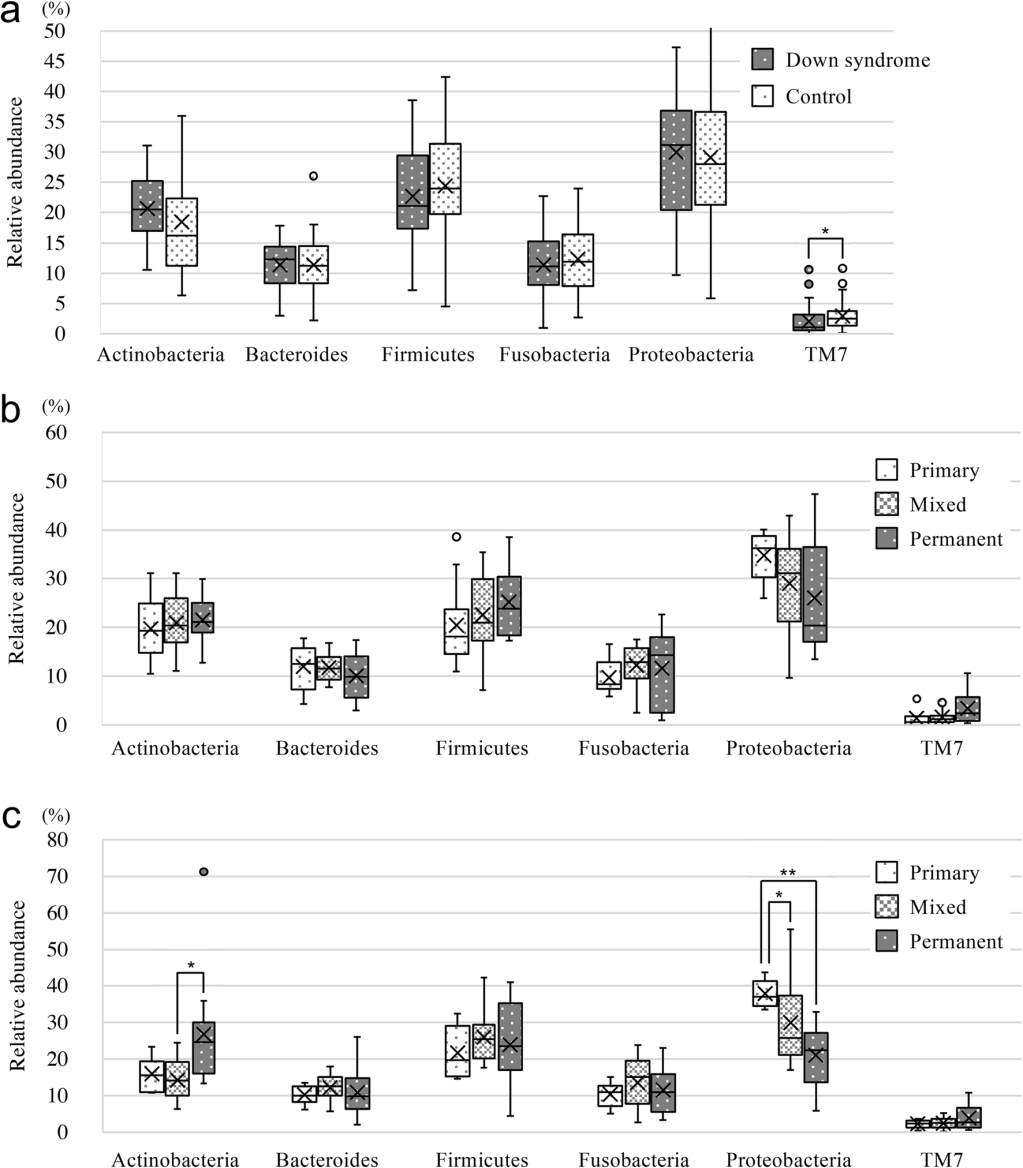
Taxonomic analysis at the phylum level in the Down syndrome and control groups. Comparisons between the two groups were made for (**a**) all participants, (**b**) Down syndrome group, and (**c**) control group. Whiskers indicate maximum and minimum values, boxes indicate the interquartile range, X indicates the mean value, and circles indicate outliers. *P < 0.05 and **P < 0.01 by the Mann–Whitney *U* test.

In both groups, 14 phyla were detected, six of which had a relative abundance of 2% or more and accounted for 95% of the total and only T7 showed a significant difference between Down syndrome and the control groups (*P* < 0.05) (Fig. 4a). Among the Down syndrome group participants, comparisons by dentition stage did not show any significant differences in relative abundance of any of the phyla (Fig. 4b). By contrast, in the control group, the relative ratios of Actinobacteria and Proteobacteria varied by dentition stage: Actinobacteria was significantly lower in mixed dentition than in permanent dentition (*P* < 0.05), and Proteobacteria was significantly lower in mixed and permanent dentition than in primary dentition (*P* < 0.05) (Fig. 4c).

### Taxonomic analysis at the genus or species level

**Figure 5:**
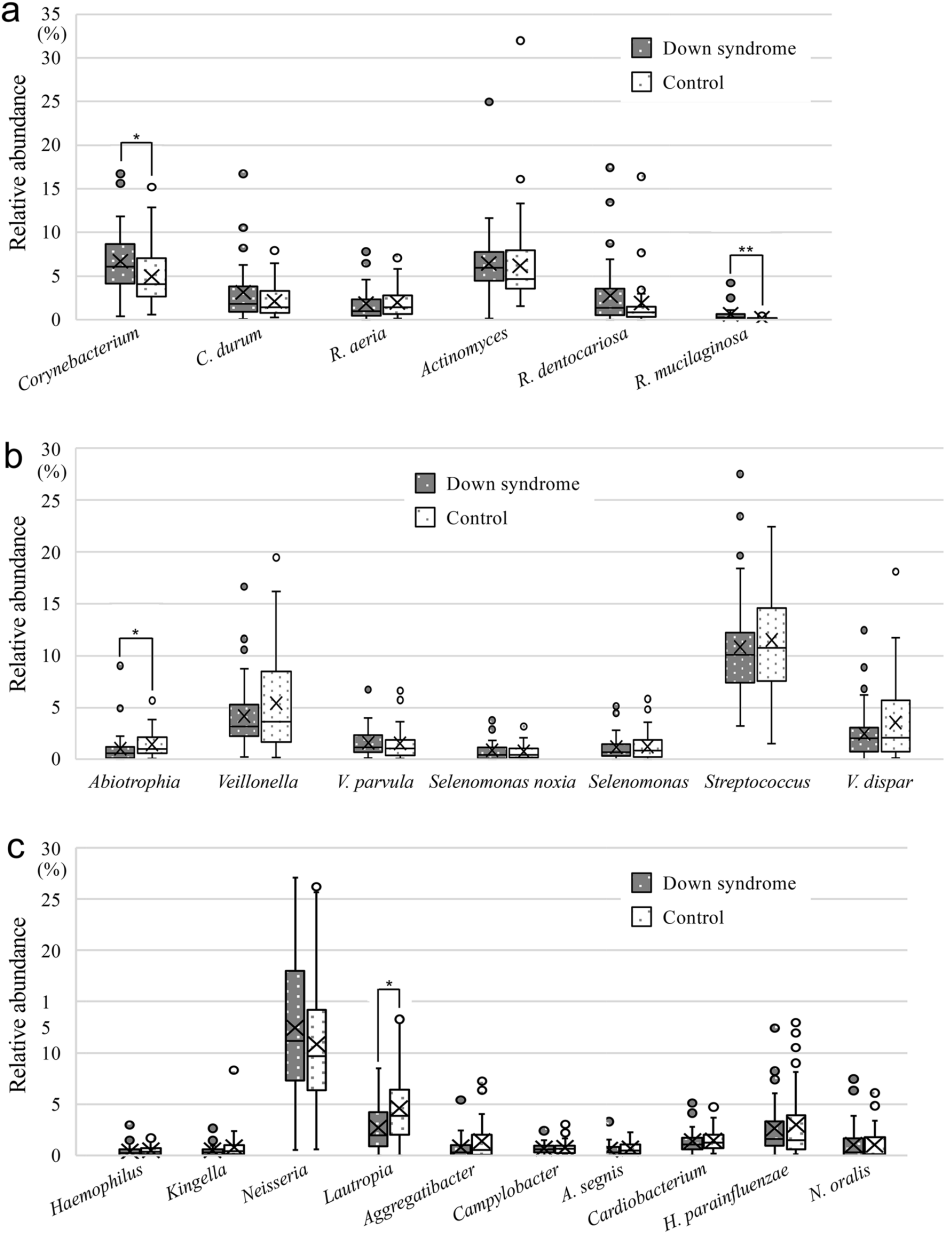
Taxonomic analysis at the genus or species level in the Down syndrome and the control groups. Taxonomic analysis of (**a**) Actinobacteria, (**b**) Firmicutes, and (**c**) Proteobacteria in the two groups. Whiskers indicate maximum and minimum values, boxes indicate the interquartile range, X indicates the mean value, and circles indicate outliers. *P < 0.05 and **P < 0.01 by the Mann–Whitney *U* test.

Taxonomic analysis detected the presence of 81 genera and 53 species in the two groups. In the phylum Actinobacteria, *Corynebacterium* and *Rothia mucilaginosa* showed significantly higher rates of relative abundance in the Down syndrome group than the control group (*P* < 0.05) (Fig. 5a). In the phylum Firmicutes, only *Abiotrophia* showed a significant difference between the two groups, with a significantly higher relative abundance in the control group (*P* < 0.05) (Fig. 5b). In the phylum Proteobacteria, *Lautropia* showed significantly higher relative abundance in the control group than in the Down syndrome group (*P* < 0.05) (Fig. 5c).

## Discussion

People with Down syndrome have specific oral findings, including higher susceptibility to periodontal disease compared with non-Down syndrome individuals^4^. To better understand the unique oral conditions in Down syndrome, it may be important to clarify the oral microbiome in childhood. Therefore, we performed a metagenomic analysis of the oral bacteria of children with and without Down syndrome to identify bacteria that may be specific to children with the syndrome.

Children with Down syndrome exhibit mental retardation and generally have lower oral cleaning ability compared with non-Down syndrome children^1^. However, we compared the plaque index between the Down syndrome and control groups and found no significant difference, suggesting that the effect of dental plaque accumulation on the oral microbiome of each group is small. Our pediatric subjects with Down syndrome undergo regular checkups at the pediatric outpatient clinic of the University Dental Hospital every 3–4 months. At each visit, these children receive professional oral cleaning and their parents are provided with instructions for daily cleaning at home. Therefore, we expect that the parents of our subjects routinely perform oral care for their children with Down syndrome, regardless of age, which may explain why there was no difference in the plaque index between the Down syndrome and non-Down syndrome groups.

In the Down syndrome group, there was a subject with periodontal pockets measuring 6 mm. The percentage of bleeding on probing, performed simultaneously with periodontal pocket measurement using a probe, was significantly higher in the Down syndrome group than in the non-Down syndrome group, consistent with a previous report^11^. Therefore, children with Down syndrome were more likely to develop periodontal pockets and bleeding from periodontal tissue, even when their plaque control was comparable to that of non-Down syndrome children.

A comparison of the numbers of OTUs detected in both groups revealed a significantly richer bacterial species constitution in the Down syndrome group. In the Faith’s phylogenetic diversity analysis, which uses an algorithm that considers the branch length of the phylogenetic tree based on the number of OTUs, the number of bacterial species was significantly higher in the Down syndrome group. Furthermore, the β-diversity analysis also revealed significant differences in the oral microbiota between the Down syndrome and the control groups. Therefore, the comparative richness in bacterial species in the Down syndrome group was not only numerical, it was phylogenetic.

Interestingly, OTU analysis of dentition stage subgroups only showed significantly higher numbers of bacterial species in participants with Down syndrome who were in the mixed dentition stage. Additionally, permutational multivariate analysis of variance (PERMANOVA) analysis showed a significant difference in β-diversity of commensal bacteria between the mixed dentition subgroups of the control and Down syndrome participants. Oral bacteria, particularly periodontal bacteria that release toxins and induce inflammation, can contribute to the exacerbation of other diseases that develop later in life, even if their presence is transient^12^. Therefore, any flora that specifically colonize the oral cavity of children with Down syndrome during the mixed dentition stage, even transiently, might also affect the pathogenicity of this syndrome.

In Down syndrome, eruption of the primary dentition is delayed, and is not complete until 4–5 years of age^13^. However, replacement of primary teeth with permanent teeth is also delayed in children with Down syndrome, with the first molars erupting at 8–9 years of age^14^. Such reports suggest that eruption of the primary and permanent teeth is delayed by a few years in children with Down syndrome; thus, the length of the mixed dentition period may not differ significantly between children with and without Down syndrome. However, because the approximate timing of colonization and infection has been identified for some oral bacteria^15^, it is possible that the delay in tooth eruption in people with Down syndrome affects their OTU number.

According to findings obtained from adults, there are no differences in the OTU number between healthy periodontal tissue and gingivitis^16^. In the present study, we focused on children between 2 and 18 years of age (mean age, 9 years) and found that the presence of gingival inflammation did not affect the OTU number in the controls or participants with Down syndrome. However, among participants with gingivitis, those with Down syndrome showed higher scores of α- and β-diversity compared with the controls. These results indicated that, during gingival inflammation, a variety of bacterial species that colonize individuals with Down syndrome are taxonomically unlike those that colonize non-Down syndrome individuals.

Both the numbers and level of functioning of immune system cells are lower in people with Down syndrome than in those without Down syndrome^17^. The specific immunological characteristics of Down syndrome may influence a high frequency of bacterial infection and the susceptibility to gingival inflammation. Furthermore, such characteristics may be related to the difference in variation in α-diversity with and without gingival inflammation between the Down syndrome and non-Down syndrome groups in this study. Therefore, analyses of the impacts of Down syndrome-specific immunological characteristics on the oral microbiota should be performed in the future.

In the phylum level analysis, six of the 14 phyla (Proteobacteria, Firmicutes, Actinobacteria, Bacteroidetes, Fusobacteria, and TM7) were the most dominant in both groups, with a relative abundance of 2% or higher. Of these six phyla, only TM7 (also known as Saccharibacteria) was significantly more abundant in the control group than the Down syndrome group. The TM7 phylum is ubiquitous in the human oral microbiome and comprises non-culturable species^18^. These bacteria possess parasitic properties and are involved in oral mucosal infections. Although a previous study implicated TM7 in the development of gingivitis and periodontitis^19^, our results indicated that TM7 has a seemingly small effect on the development of periodontal disease in Down syndrome.

Of the six most abundant phyla in the control group, Actinobacteria was comparatively less abundant during primary and mixed dentition and more abundant during permanent dentition, whereas the abundance of Proteobacteria decreased with the progression of dentition. By contrast, in the Down syndrome group, both Actinobacteria and Proteobacteria showed high abundance at all three dental stages. Actinobacteria are associated with healthy periodontal tissue in adults^16,19^, while Proteobacteria comprise commensal bacteria of the oral cavity, skin and gastrointestinal tract^20^. Although neither phylum includes any major pathogens of periodontal disease, colonization of bacteria with low periodontal pathogenicity in childhood is important for the establishment of highly pathogenic bacteria later in life^15^. It has also been reported that children or adults with Down syndrome have a lower occurrence of dental caries^21,22^, and this held true for the permanent dentition participants in our study. Therefore, the presence of certain bacteria, including Actinobacteria and Proteobacteria, in children with Down syndrome may affect the development of dental caries and periodontal disease specific to the syndrome.

The oral health status of children still varies greatly among countries, even within Asia. For example, the oral conditions of children in Cambodia and Japan are very different^23,24^. Our study is the first to focus on the microbiome of Down syndrome, and we analyzed only oral samples collected from Japanese children. In the future, whether the oral microflora unique to children with Down syndrome identified in this study is specific to Japanese children or common to children in other countries should be clarified.

We analyzed the oral microbiomes in children with and without Down syndrome. Our results showed that red complex species (*Porphyromonas*, *Treponema*, and *Tannerella*), known as highly periodontopathic bacteria, were not detectable in children with Down syndrome. In contrast, Nóvoa et al.^7^ analyzed the oral microbiome using a method similar to ours, but focused only on adults (> 18 years) with Down syndrome. They found that the red complex species were significantly more abundant in people with Down syndrome and periodontitis than in people with Down syndrome without periodontal disease. These results suggest that periodontopathic bacteria with high pathogenicity colonize the oral cavity later in adulthood in people with Down syndrome and are involved in the exacerbation of periodontal disease.

Okada et al.^25^ detected *Porphyromonas gingivalis* and *Aggregatibacter actinomycetemcomitans* in samples from children that were collected by brushing with a sterile toothbrush. Because our study included younger children with Down syndrome who were difficult to manage, we decided to use their safe, simple method^25^ for sample collection. However, we recognize that, for more detailed analysis of periodontopathic bacterial species, it is preferable to collect subgingival plaque using a scaler or curette, and methods using these tools should be considered for future studies.

Although we identified oral bacteria specific to children with Down syndrome, this study had certain limitations. First, our study had a small sample size. Similar studies with larger sample sizes should be performed to confirm the applicability of our results to all children with Down syndrome. Additionally, sequencing of the V4 hypervariable region of 16S rRNA has low resolution at the genus level (and below). Furthermore, the roles of each of the bacterial species that were significantly more abundant in the Down syndrome group are unclear. A more detailed analysis of each bacterial species should be undertaken.

In summary, the oral microbiome of participants with Down syndrome markedly differed from that of the controls, and was primarily observed in participants with mixed dentition and gingivitis. Our results suggest that the oral microbiome of children with Down syndrome may influence the oral diseases specific to this syndrome.

## Methods

### Ethic statement

This study was conducted in compliance with the Declaration of Helsinki. This study was approved by the Independent Ethics Committee of Hiroshima University Hospital, Hiroshima, Japan (No. E-D-56-5). All study participants and their legal guardians provided written informed consent before study enrollment.

### Study population

The age distribution and oral condition of participants are shown in Table 1. Forty patients with Down syndrome (18 boys and 22 girls; mean age, 10.20 ± 4.45 years; range, 2–18 years) who received the consent of the researcher from the pediatric outpatient clinic of Hiroshima University Dental Hospital were enrolled into the Down syndrome group. Forty children without Down syndrome (18 boys and 22 girls; mean age, 9.31 ± 4.34 years; range, 2–18 years), who were selected on the basis of scores on the age-based DMFT/dmft (for primary teeth) index from the 2016 Ministry of Health, Labor and Welfare Survey on Dental Diseases, were enrolled into the control group. Participants had not taken an antibacterial drug within 1 month and had not eaten or drank anything for at least 1 h prior to the examination, and at least 2 h had passed since the last tooth brushing.

### Oral examination

The oral health characteristics of the participants are shown in Table 2. Plaque adhesion status was assessed using the plaque index (PLI), gingival inflammation was measured using the GI, and gingival cleft status was evaluated using the probing depth (PD)^9,26,27^. For the three examinations listed above, the upper right first molar, upper left lateral incisor, upper left first premolar, lower right lateral incisor, lower right first premolar and lower left first molar (or the upper right primary second molar, upper left primary lateral incisor, upper left primary first molar, lower right primary lateral incisor, lower right primary first molar, and lower left primary second molar when the tooth was a primary tooth) were examined, and the highest value obtained was used as the score for each participant. The presence of BOP in the same tooth area was also examined, and considered to be positive even with one bleeding point. Using GI evaluation criteria, children with GI scores of 0 or 1 were classified into the non-inflamed group, while those with a GI score of 2 were classified into the inflamed group. All examinations were performed by pediatric dentists from our department.

### Sample collection and DNA extraction

Dental plaque samples were obtained according to the method of Okada et al.^25^. After brushing with a sterile toothbrush for 1 min, attached dental plaque samples were immersed in 15 mL sterile saline, centrifuged at 5000 rpm for 15 min, and the pellets were stored at − 80 °C until DNA purification. DNA was extracted from samples using the Zymo BIOMICS DNA mini kit (Zymo Research, Irvine, CA, USA) according to the manufacturer’s protocol.

### Library preparation

Amplification of the V4 hypervariable region of 16S rRNA was performed by PCR using KAPA HiFi HotStart ReadyMix (KAPA Biosystems, Wilmington, MA, USA) and Nextera XT V4 index primers (Forward: TCG TCG GCA GCG TCA GAT GTG TAT AAG AGA CAG GTG YCA GCM GCC GCG GTA A; Reverse: GTC TCG TGG GCT CGG AGA TGT GTA TAA GAG ACA GGG ACT ACH VGG GTW TCT AAT) (Illumina, San Diego, CA, USA). PCR cycling was performed as follows: initial denaturation at 95 °C for 3 min; 25 cycles of 95 °C for 30 s, 55 °C for 30 s, and 72 °C for 30 s; and a final extension at 72 °C for 5 min. PCR reactions were analyzed by electrophoresis on a 2% agarose gel to confirm the presence of a ~ 330-bp amplification product.

PCR products were purified using Agencourt AMPure XP magnetic beads (Beckman Coulter, Brea, CA, USA). Barcode sequence addition was performed by PCR using the Nextera XT Index Kit (Illumina) and Nextera XT index primers. PCR cycling was performed as follows: initial denaturation at 95 °C for 3 min; 8 cycles of 95 °C for 30 s, 55 °C for 30 s, and 72 °C for 30 s; and a final extension at 72 °C for 5 min. Primers used in this PCR reaction, including adapter sequences, are shown in Supplementary Table 1. The amplified product was confirmed to be ~ 460 bp on a Bioanalyzer 2100 (Agilent Technologies, Santa Clara, CA, USA), and was re-purified using Agencourt AMPure XP magnetic beads.

Samples were quantified using KAPA SYBR FAST qPCR MasterMix and Primer Premix (KAPA Biosystems) and Applied Biosystems 7900HT software (Thermo Fisher Scientific). PCR cycling was performed as follows: 35 cycles of 95 °C for 5 min, 95 °C for 30 s, and 60 °C for 45 s. The concentration of amplification products was measured using an Invitrogen Qubit 3.0 Fluorometer (Thermo Fisher Scientific) and a Bioanalyzer 2100 (Agilent Technologies).

### 16S rRNA gene sequencing and OTU analysis

The library concentration was adjusted to 4 nM with Qiagen EB Buffer and mixed in one tube. The Miseq platform system (Illumina) was employed with 25% Phix as a control.

The quality of the leads obtained from all samples was checked by FastQC (http://www.bioinformatics.babraham.ac.uk/projects/fastqc/) and only reads considered to be appropriate for analyses were examined. An OTU table was created using Dada2 (http://www.ncbi.nlm.nih.gov/pmc/articles/PMC4927377/). Qiime2 was used for the diversity analysis and taxonomic classification, while the Naïve Bayes classifier (Greengenes13_8 99% OTU full-length sequence) was employed for only taxonomic classification. The phylum, genus, and species were extracted from taxa with relative abundance > 1% of the total in the Down syndrome or non-Down syndrome groups, according to the method previously described^28^. All findings related to bacterial phyla, genera, and species obtained from microbiome analysis were validated and polished using the eHOMD (Human Oral Microbiome D) and LPSN databases.

### Statistical analysis

The Mann–Whitney *U* test was used to analyze the DMFT/dmft index, PLI, GI and PD data. The Chi-square test of independence (Fisher’s exact probability test for dentition comparisons) was employed to compare the prevalence rates of caries and BOP. A significance level of *P* < 0.05 was applied to all results. A rarefaction analysis was performed for the α- and β-diversity analyses, and the sequence depth was adjusted to the sample with the smallest number of reads (approximately 56,000 reads). Faith’s phylogenetic diversity and principal coordinate analysis of the unweighted UniFrac distance were performed in α- and β-diversity analyses, respectively. Regarding significance testing, the Kruskal–Wallis test and the PERMANOVA were used for α-diversity and β-diversity, respectively. The Mann–Whitney *U* test was conducted to analyze the relative abundances of the constituent bacteria.

## Supplementary Information

Supplementary Table 1.

## Data Availability

Sequence data have been deposited at the BioSample database of the DNA Data Bank of Japan (DDBJ) under accession number DRA014015.
