# Supplementary material for: Characterization of the unique oral microbiome of children with Down syndrome
Source: Sci Rep. 2022 Aug 19;12:14150. doi: 10.1038/s41598-022-18409-z (PMC9391446; doi:10.1038/s41598-022-18409-z)
Supplement: Supplementary file 1 — Supplementary Table 1. [file 41598_2022_18409_MOESM1_ESM.pdf]

Characterization of the unique oral microbiome of children with Down syndrome

Chieko Mitsuhata, Nao Kado, Masakazu Hamada, Ryota Nomura & Katsuyuki Kozai

Supplementary Table 1 List of Nextera XT index primer.

N7XX	Sequence	S5XX	Sequence
N701	TAAGGCGA	S502	CTCTCTAT
N702	CGTACTAG	S503	TATCCTCT
N703	AGGCAGAA	S504	AGAGTAGA
N704	TCCTGAGC	S505	GTAAGGAG
N705	GGACTCCT	S506	ACTGCATA
N706	TAGGCATG	S507	AAGGAGTA
N707	CTCTCTAC	S508	CTAAGCCT
N708	CAGAGAGG	S517	GCGTAAGA
N710	CGAGGCTG		
N711	AAGAGGCA		
